# Dual Actions of A_2A_ and A_3_ Adenosine Receptor Ligand Prevents Obstruction-Induced Kidney Fibrosis in Mice

**DOI:** 10.3390/ijms22115667

**Published:** 2021-05-26

**Authors:** Eun Seon Pak, Lak Shin Jeong, Xiyan Hou, Sushil K. Tripathi, Jiyoun Lee, Hunjoo Ha

**Affiliations:** 1 Graduate School of Pharmaceutical Sciences, College of Pharmacy, Ewha Womans University, Seoul 03760, Korea; louisa9419@gmail.com; 2 Future Medicine Co., Ltd., Seongnam 13449, Korea; lakjeong@snu.ac.kr; 3 Department of Pharmacy, College of Pharmacy, Seoul National University, Seoul 08826, Korea; xyhous@gmail.com (X.H.); bipinsu.5@gmail.com (S.K.T.)

**Keywords:** chronic kidney disease, fibrosis, inflammation, adenosine, adenosine receptors

## Abstract

Kidney fibrosis is the final outcome of chronic kidney disease (CKD). Adenosine plays a significant role in protection against cellular damage by activating four subtypes of adenosine receptors (ARs), A_1_AR, A_2A_AR, A_2B_AR, and A_3_AR. A_2A_AR agonists protect against inflammation, and A_3_AR antagonists effectively inhibit the formation of fibrosis. Here, we showed for the first time that LJ-4459, a newly synthesized dual-acting ligand that is an A_2A_AR agonist and an A_3_AR antagonist, prevents the progression of tubulointerstitial fibrosis. Unilateral ureteral obstruction (UUO) surgery was performed on 6-week-old male C57BL/6 mice. LJ-4459 (1 and 10 mg/kg) was orally administered for 7 days, started at 1 day before UUO surgery. Pretreatment with LJ-4459 improved kidney morphology and prevented the progression of tubular injury as shown by decreases in urinary kidney injury molecular-1 (KIM-1) and neutrophil gelatinase-associated lipocalin (NGAL) excretion. Obstruction-induced tubulointerstitial fibrosis was attenuated by LJ-4459, as shown by a decrease in fibrotic protein expression in the kidney. LJ-4459 also inhibited inflammation and oxidative stress in the obstructed kidney, with reduced macrophage infiltration, reduced levels of pro-inflammatory cytokines, as well as reduced levels of reactive oxygen species (ROS). These data demonstrate that LJ-4459 has potential as a therapeutic agent against the progression of tubulointerstitial fibrosis.

## 1. Introduction

Kidney fibrosis is characterized by glomerulosclerosis, vascular sclerosis, and tubulointerstitial fibrosis and is considered the final outcome of chronic kidney disease (CKD) [1,2]. As the tubulointerstitial space occupies more than 90% of the kidney, tubulointerstitial fibrosis is an easily observed pathological process leading to end-stage kidney disease (ESKD) [3]. Tubulointerstitial fibrosis is accompanied by: (i) the infiltration of inflammatory cells, (ii) the activation of fibroblasts, (iii) the accumulation of extracellular matrix (ECM), and (iv) the production of tubular atrophy [4]. These events occur partially or together.

Adenosine is formed both intracellularly and extracellularly through catalytic enzymes that hydrolyze nucleotides [5]. Under distress conditions, including ischemia, hypoxia, and inflammation, extracellular ATP levels are increased and rapidly hydrolyzed to adenosine. Adenosine serves as a signaling molecule that initiates receptor-mediated functions. The adenosine receptors (ARs) are named adenosine receptor A_1_ (A_1_AR), adenosine receptor A_2A_ (A_2A_AR), adenosine receptor A_2B_ (A_2B_AR), and adenosine receptor A_3_ (A_3_AR) [6]. The four ARs belong to the family of G protein coupled receptors (GPCRs), and ARs signaling occurs not only through inhibition or stimulation of adenylyl cyclase (cAMP), but also through phospholipase C (PLC), Ca^2+^, and mitogen-activated protein kinases (MAPKs) [7].

In the kidney, adenosine regulates the kidney physiological responses such as tubuloglomerular feedback (TGF), blood flow, glomerular filtration rate (GFR), renin release, and NaCl transport [8,9]. However, a chronically excessive kidney adenosine level causes tubulointerstitial fibrosis and kidney dysfunction [10,11]. 

Interestingly, 8-(p-sulfophenyl) theophylline, a non-selective ARs antagonist, effectively decreased kidney fibrosis and improved kidney function [11]. Our previous studies have demonstrated that novel, orally active, species-independent, A_3_AR antagonists protected against UUO-induced kidney fibrosis [12] and diabetic nephropathy [13] by modulating ECM accumulation and fibroblast activation. Renoprotective effects of pharmacological inhibition [14,15,16,17] and genetic deficiency of A_3_AR [15] have been reported in several kidney injuries including ischemia-reperfusion injury (IRI) [14,15], myoglobinuria injury [15], and adriamycin-induced nephropathy [17]. Besides, A_2A_AR acts as a strong anti-inflammatory effector responding to extracellular adenosine [18]. 

Numerous studies have demonstrated that A_2A_AR is expressed in inflammatory and immune cells, such as monocyte [19], neutrophils [20], lymphocytes [21], and NK cells [22]. Pharmacological activation of A_2A_AR [23,24,25,26,27,28,29,30] has been shown to be effective in several kidney injury models such as IRI [23,24,25], diabetic kidney injury [26,27], puromycin-induced podocyte injury [28], and UUO-induced kidney fibrosis [29,30]. These results provide evidence of dual action as an A_3_AR antagonist, and a A_2A_AR agonist may have better protective effects against kidney injuries. 

Thus, the present study has determined the renoprotective effect of LJ-4459, a newly developed potent dual acting A_2A_ and A_3_ AR ligand on UUO-induced tubulointerstitial fibrosis. LJ-4459 has been reported (i) to have high binding affinity to both hA_2A_AR and hA_3_AR, (ii) to be a full A_2A_AR agonist and a full competitive A_3_AR antagonist, (iii) to have 0.51 of log P, and (iv) to have similar anti-inflammatory potency as indomethacin in carrageenan-induced paw edema assay [31].

## 2. Results

### 2.1. Pretreatment of LJ-4459 Improves Kidney Function and Attenuates Kidney Tubular Injury in the Obstructed Kidney

**Figure 1:**
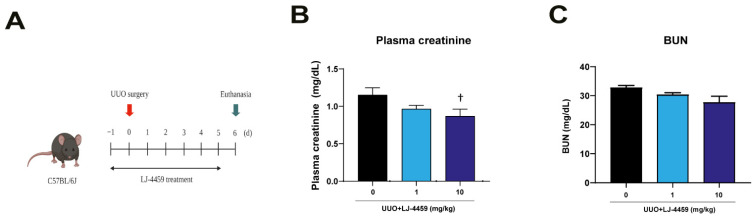
LJ-4459 improves kidney function in UUO mice. (**A**) Schematic diagram of the in vivo experimental schedule. Drug administration was start at 1 d before the UUO surgery, and mice were euthanized after 7 d of treatment. Plasma was analyzed for (**B**) plasma creatinine (mg/dL) and (**C**) BUN (mg/dL). Data are presented as mean ± SE of 7–8 mice. † *p* < 0.05 vs. UUO mice.

**Figure 2:**
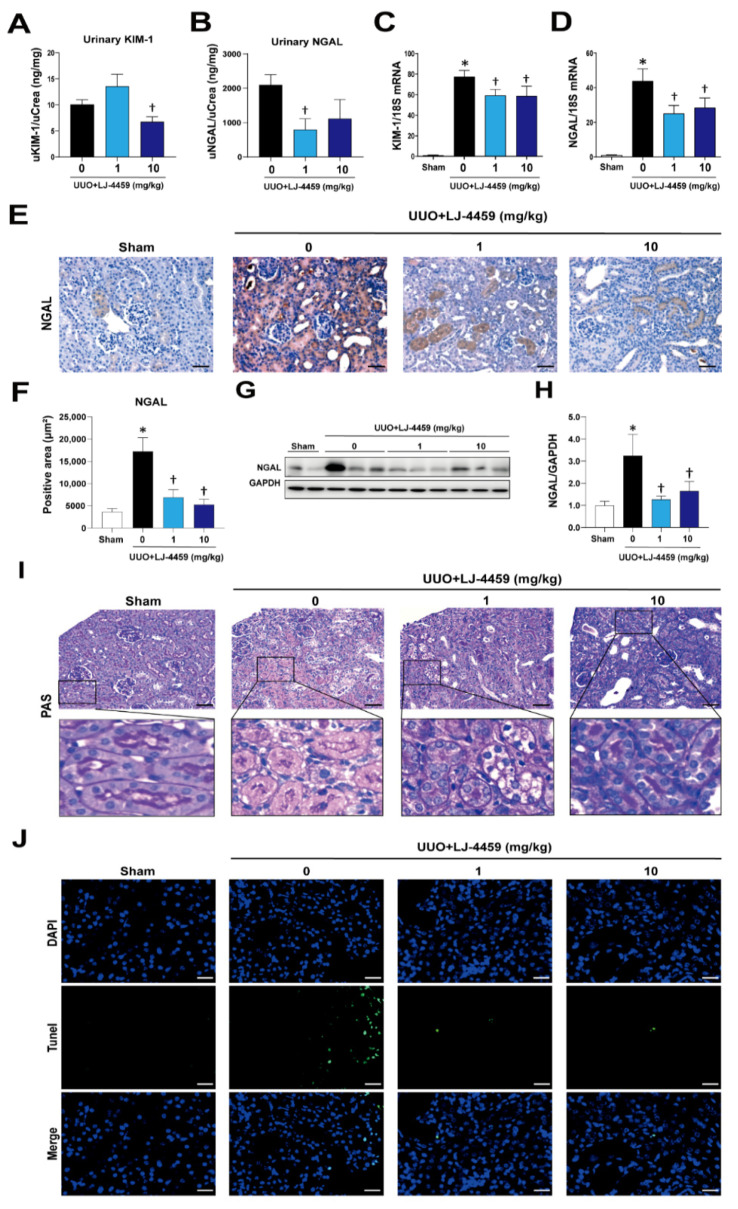
LJ-4459 attenuates kidney tubular injury in UUO mice. Urine was analyzed for (**A**) urinary KIM-1 (ng/mg), and (**B**) urinary NGAL (ng/mg). (**C**) The mRNA levels of KIM-1 were measured. (**D**) The mRNA levels of NGAL were measured. Levels of mRNA were normalized with 18S. Paraffin-embedded kidney sections were stained with (**E**,**F**) NGAL antibody (1:200; original magnification: 200×; scale bar: 50 μm). (**G**,**H**) Immunoblotting analysis of NGAL in the kidney. The levels of proteins were normalized with GAPDH. (**I**) Paraffin-embedded kidney sections were stained with PAS (original magnification: 200×; scale bar: 50 μm: enlarged images have been shown in the inset). (**J**) TUNEL assay (original magnification: 400×; scale bar: 20 μm). Data are presented as mean ± SE of 7–8 mice. * *p* < 0.05 vs. sham mice, † *p* < 0.05 vs. UUO mice.

Our previous studies have established an obstruction-induced tubulointerstitial fibrosis model in rat and mice [12,32]. Based on these analyses, mice were administered LJ-4459 to start 1 d prior to UUO surgery and all mice were euthanized after 7 d of treatment (Figure 1A). The urinary adenosine excretion was significantly increased after UUO surgery (data not shown), which was not affected by LJ-4459 treatment (Supplementary Figure S1). We examined the effect of LJ-4459 on kidney dysfunction and kidney tubular injury in the obstructed kidneys. Plasma creatinine and blood urea nitrogen (BUN), markers of kidney injury, were significantly increased in UUO mice [32]. Plasma creatinine was effectively reduced by 10 mg/kg LJ-4459 treatment (Figure 1B). BUN tended to be decreased by LJ-4459 treatment, but it did not reach statistical significance (Figure 1C). In addition, kidney tubular injury markers such as urinary KIM-1 and NGAL were significantly reduced by 10 and 1 mg/kg treatment with LJ-4459, respectively (Figure 2A,B). The KIM-1 mRNA levels increased in the obstructed kidneys, which were reduced by LJ-4459 treatment (Figure 2C). The NGAL mRNA and protein expression levels increased after UUO surgery and were decreased by LJ-4459 treatment (Figure 2D–H). The results of PAS staining showed morphology changes as indicated by tubular atrophy after UUO surgery, which was attenuated by LJ-4459 treatment (Figure 2I). Tubular cell apoptosis indicated by TUNEL staining was increased in the obstructed kidneys, which was decreased by LJ-4459 treatment (Figure 2J). To examine the toxicity of LJ-4459, we performed the MTT assay using mouse proximal tubular epithelial (mProx) cells. LJ-4459 did not affect cell viability up to 100 μM (Supplementary Figure S2), suggesting lack of toxicity. 

### 2.2. Pretreatment of LJ-4459 Decreases Kidney Inflammation in the Obstructed Kidney

**Figure 3:**
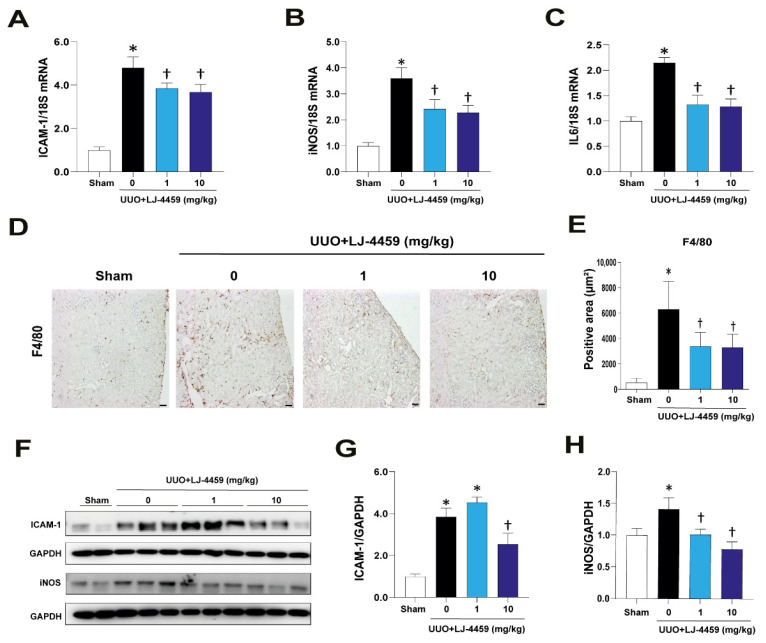
LJ-4459 ameliorates kidney inflammation in UUO mice. (**A**–**C**) The mRNA levels of inflammation markers such as ICAM-1, iNOS, and IL-6 were measured by real time RT-PCR. The levels of mRNA were normalized with 18S. (**D**,**E**) Paraffin-embedded kidney sections were stained with anti-F4/80 antibody (1:400; original magnification: 100×; scale bar: 50 μm). Immunoblotting analysis of (**F**–**H**) ICAM-1 and iNOS in the kidney. The levels of proteins were normalized with GAPDH. Data are presented as mean ± SE of 7–8 mice. * *p* < 0.05 vs. sham mice, † *p* < 0.05 vs. UUO mice.

We evaluated the anti-inflammatory effect of LJ-4459 in the obstructed kidneys. As expected, the obstructed kidneys led to an increase of inflammatory cytokines such as ICAM-1, iNOS, and IL-6, which were reduced in response to LJ-4459 treatment (Figure 3A–C). Macrophage infiltration, as indicated by F4/80-positive staining, was increased in the obstructed kidneys, whereas LJ-4459-treated obstructed kidneys had decreases in these effects (Figure 3D,E). In parallel, the protein expression levels of ICAM-1 and iNOS were increased in the obstructed kidneys, which were reduced by LJ-4459 treatment (Figure 3F–H). 

### 2.3. Pretreatment with LJ-4459 Inhibits Tubulointerstitial Fibrosis in the Obstructed Kidney

**Figure 4:**
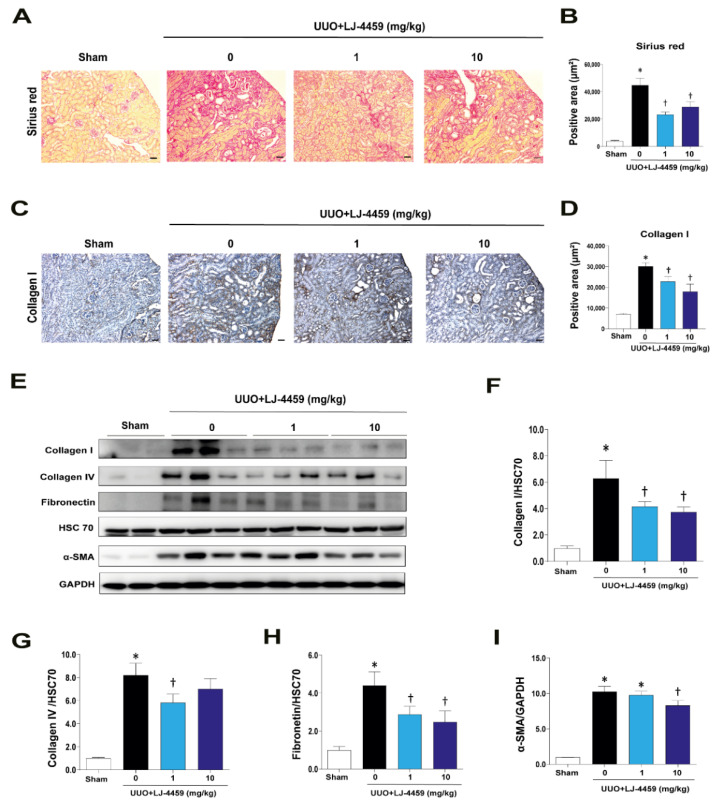
LJ-4459 inhibits kidney fibrosis in UUO mice. Paraffin-embedded kidney sections were stained with (**A**,**B**) sirius red staining (original magnification: 100×; scale bar: 50 μm) and (**C**,**D**) anti-collagen I antibody (1:400; original magnification: 100×; scale bar: 50 μm). (**E**–**I**) Immunoblotting analysis of collagen I, collagen IV, fibronectin, and α-SMA in the kidney. The levels of proteins were normalized with HSC70 or GAPDH. Data are presented as mean ± SE of 7–8 mice. * *p* < 0.05 vs. sham mice, † *p* < 0.05 vs. UUO mice.

In order to confirm whether LJ-4459 has an anti-fibrotic effect, we detected the ECM proteins, such as collagen I, collagen IV, and fibronectin, and α-smooth muscle actin (α-SMA). Picrosirius red staining indicated that there was a lot of UUO-induced accumulation of ECM in the obstructed kidneys. The excessive accumulation of ECM was reduced after LJ-4459 treatment (Figure 4A,B). The collagen I staining results showed that positively stained areas were increased in the obstructed kidneys and were decreased in LJ-4459-treated obstructed kidneys (Figure 4C,D). Consistently, the collagen I, collagen IV, fibronectin, and α-SMA protein expression levels were significantly increased in obstructive kidneys. These ECM proteins and α-SMA expression levels were inhibited by LJ-4459 treatment (Figure 4E–I). 

### 2.4. Pretreatment with LJ-4459 Reduces Oxidative Stress in the Obstructed Kidney

**Figure 5:**
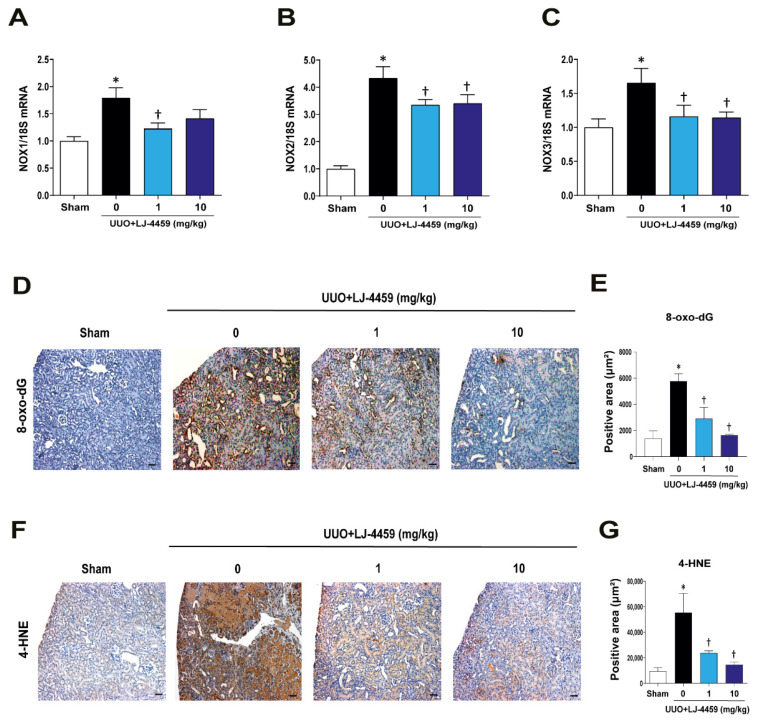
LJ-4459 decreases oxidative stress in UUO mice. (**A**–**C**) The mRNA levels of oxidative markers such as NOX1, NOX2, and NOX3 were measured by real time RT-PCR. The levels of mRNA were normalized with 18S. Paraffin-embedded kidney sections were stained with (**D**,**E**) 8-oxo-dG antibody (1:400; original magnification: 100×; scale bar: 50 μm) and (**F**,**G**) anti-4HNE antibody (1:200; original magnification: 100×; scale bar: 50 μm). Data are presented as mean ± SE of 7–8 mice. * *p* < 0.05 vs. sham mice, † *p* < 0.05 vs. UUO mice.

We evaluated the state of oxidative stress in the obstructed kidneys. The mRNA expression levels of NOX1, NOX2, and NOX3 were increased in the obstructed kidneys. These mRNA expression levels were reduced by LJ-4459 treatment (Figure 5A–C). The 8-oxo-dG staining results showed that positively stained areas were increased in obstructed kidneys. These positively stained areas were reduced in LJ-4459-treated obstructed kidneys (Figure 5D,E). The 4-hydroxynonenal (4-HNE) staining, which indicates oxidative stress, was increased in the obstructed kidneys, whereas LJ-4459-treated obstructed kidneys had decreases in these effects (Figure 5F,G). 

### 2.5. Pretreatment with LJ-4459 Suppresses ERK and NF-κB Phosphorylation in the Obstructed Kidney

**Figure 6:**
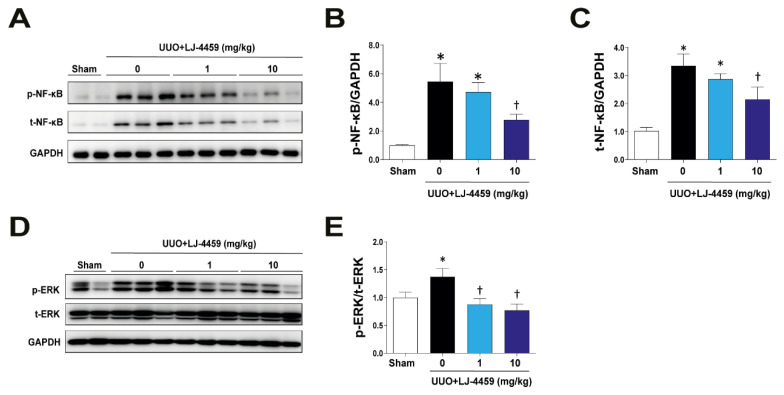
LJ-4459 downregulates ERK and NF-κB phosphorylation. (**A–E**) Immunoblotting analysis of NF-κB and ERK phosphorylation in the kidney. The levels of proteins were normalized with GAPDH or their respective total protein. Data are presented as mean ± SE of 7–8 mice. * *p* < 0.05 vs. sham mice, † *p* < 0.05 vs. UUO mice.

To dissect the mechanisms involved in the renoprotective effect of LJ-4459, we determined that the expression levels of ERK and NF-κB. ERK [12] and NF-kB [17] have been shown to mediate A_3_AR-induced kidney injury. The obstructed kidneys were markedly increased in total (t-NF-κB) expression levels and the phosphorylation of NF-κB (p-NF-κB). UUO-induced increases in total and phosphorylation of NF-κB were suppressed by LJ-4459 treatment (Figure 6A–C). In addition, phosphorylation of ERK (p-ERK) expression levels was upregulated in obstructed kidneys, which was decreased by LJ-4459 treatment (Figure 6D,E).

## 3. Discussion

The present data demonstrated that LJ-4459, a newly developed dual-acting ligand acting as both an A_2A_AR agonist and an A_3_AR antagonist [31], attenuated the progression of tubulointerstitial fibrosis in UUO mice. In addition, kidney injuries including kidney dysfunction and inflammation were improved by LJ-4459 treatment. 

Kidney fibrosis is a consequence of multiple mechanisms, including the infiltration of inflammatory cells, the production of fibrotic cytokines and growth factors, and the deposition of ECM [4,33]. In addition, ROS regulate various signaling pathways, leading to inflammation and fibrosis [34]. UUO has been used as a tubulointerstitial fibrosis model characterized by the excessive accumulation of matrix protein, degradation of the proximal tubular mass, and increased tubular cell death [35,36]. In our experimental condition, we confirmed that the UUO operation leads to kidney injury, including tubulointerstitial fibrosis (Figure 4), tubular injury (Figure 2), inflammation (Figure 3), and oxidative stress (Figure 5). 

ARs are a family of GPCRs widely distributed in almost all organs, playing roles in physiological and pathological functions by mediating downstream signaling [37]. All ARs affect cAMP levels, and the Gi-coupled A_1_AR and A_3_AR inhibit AC activity. On the other hand, the Gs-coupled A_2A_AR and A_2B_AR stimulate AC activity. Furthermore, this leads to activation of cAMP-dependent protein kinase A (PKA), MAPKs, phospholipase C (PLC), and calcium-dependent protein kinases (PKC) pathways [6,7]. 

Either A_2A_AR activation [23,24,25,26,27,28,29,30] or A_3_AR inhibition [12,13,14,15,16,17] have renoprotective effects. In particular, A_2A_AR agonists, ATL-146e [24,25] and CGS21680 [27,29,30], protect against kidney injury by decreasing cytokine expression and macrophage infiltration. In addition, A_3_AR antagonists, MRS1220 [16], LJ-1888 [12], and LJ-2698 [13], protect against kidney fibrosis via decreasing profibrotic gene expression. Interestingly, our previous study showed that an A_3_AR antagonist, LJ-2698, significantly increased A_2A_AR expression in the mouse kidney [13]. Thus, we hypothesized that as a targeted dual-acting ligand as an A_2A_AR agonist and an A_3_AR antagonist, LJ-4459, would be protective against kidney injury. As expected, LJ-4459 effectively reduced all parameters related to the progression of kidney injury, including tubular damage (Figure 2), inflammation (Figure 3), tubulointerstitial fibrosis (Figure 4), and oxidative stress (Figure 5). LJ-4459 at 1 or 10 mg/kg showed kidney protective effect in most, if not all, parameters measured in the present study. To provide full dose–response effect, it is necessary to perform experiments using a wider dosage range, including lower doses of LJ-4459. Considering the clinical implications, further studies that examine the effect of LJ-4459 on various models of CKD including diabetic kidney disease are needed.

In our present study, we showed that phosphorylation of ERK and NF-κB were increased in obstructed kidneys, and this was attenuated by LJ-4459 treatment (Figure 6). Previous studies have shown that NF-κB is a target gene of ERK signaling [38] and it contributes to kidney fibrosis [39]. In line with our data, A_2A_AR agonists have anti-inflammatory effects through the decreased phosphorylation of ERK [40,41] and NF-κB [41] in neutrophils and T cells. In addition, an A_3_AR antagonist blocked fibrosis via decreased phosphorylation of ERK [12] and NF-κB [17]. Therefore, these results suggest that the renoprotective effects of LJ-4459 are associated with the ERK and NF-κB signaling pathways. 

In contrast, A_2A_AR agonists increased phosphorylation of ERK in liver [42] and brain [43] tissue. In addition, A_3_AR antagonists increased phosphorylation of NF-κB in microglial cells [44] and mesothelial cells [45]. More importantly, an A_3_AR agonist has been shown to protect against sepsis kidney injury [46]. These controversial effects suggest that ARs mediate different roles in tissue and cell types. Moreover, the ARs have been affected by acute versus chronic diseases [9,47]. The detailed mechanism of the effect of LJ-4459 on A_2A_AR and A_3_AR in CKD is not clear yet.

In summary, LJ-4459, a new dual-acting agent that acts as both an A_2A_AR agonist and an A_3_AR antagonist, effectively prevented obstruction-induced kidney dysfunction, inflammation, tubulointerstitial fibrosis, and oxidative stress. 

## 4. Materials and Methods

### 4.1. Reagents

All chemicals and reagents were purchased from Sigma-Aldrich (St. Louis, MO, USA) unless otherwise specified.

### 4.2. Animal Experiments

All experimental animals were approved by the Institutional Animal Care and Use Committee at Ewha Womans University (IACUC No. 18-007, 9 March 2018). The 6-week-old male C57BL/6J mice were purchased from the Ewha Laboratory Animal Genomic center (Seoul, Korea). Unilateral ureteral obstruction (UUO) surgery was performed as described in our previous study [12,32]. Mice were housed in a room maintained at 22 ± 2 °C with a 12 h dark/12 h light cycle and were randomly divided into three groups: (i) UUO without LJ-4459 treatment (0 mg/kg), (ii) UUO+LJ-4459 1 mg/kg, and (iii) UUO+LJ-4459 10 mg/kg. Briefly, to creative the kidney tubulointerstitial fibrosis model, the left ureter was ligated at two points with silk (4-0; Ailee Co., Ltd., Busan, Korea) and was cut between the two ligation points. The UUO mice were administered 0.25% carboxymethyl cellulose (CMC) or LJ-4459 (1 or 10 mg/kg) for 7 d by oral gavage. Drug administration was start at 1 d before the UUO surgery, and all mice were euthanized after 7 d of treatment. Contralateral kidney of UUO without LJ-4459 treatment was used as a sham kidney.

### 4.3. Measurements of Blood Parameters

Blood was collected from the jugular vein before sacrifice and centrifuged at 3000 rpm for 15 min at 4 °C to collect the serum from the supernatant. Plasma creatinine (Arbor Assays, Ann Arbor, MI, USA) and blood urea nitrogen (BUN, Arbor Assays, Ann Arbor, MI, USA) were measured by using ELISA kits. 

### 4.4. Measurements of Urine Parameters

Urine was collected in metabolic cage for 24 h and centrifuged at 3000 rpm for 15 min at 4 °C. Urinary kidney injury molecular-1 (KIM-1, MKM100, R&D Systems, Minneapolis, MN, USA) and urinary neutrophil gelatinase-associated lipocalin (NGAL, Immunology Consultants Laboratory, Inc., Portland, OR, USA) were measured by using ELISA kits. 

### 4.5. Histology and Immunohistochemistry

The kidney was fixed with 4% paraformaldehyde-lysine-periodate (pH 7.4), dehydrated, embedded in paraffin, and sectioned. To examine the kidney morphology, 3 μm tissue sections were stained with periodic acid–Schiff (PAS, Abcam, Cambridge, MA, USA) reagent. To examine the kidney collagen accumulation in the kidney, 5 μm tissue sections were stained with picrosirius red (Abcam) reagent. Immunohistochemistry used anti-neutrophil gelatinase-associated lipocalin (NGAL, 1:200; Abcam), anti-F4/80 (1:400; Santa Cruz Biotechnology, Inc., Santa Cruz, CA, USA), anti-8-hydroxy-2-deoxyguanosine (8-oxo-dG, 1:400; Trevigen, Gaithersburg, MD), anti-4-hydroxynonenal (4-HNE, 1:200; Nikken SEIL Co., Shizuoka, Japan), and anti-collagen I (1:400; Southern Biotech, Birmingham, CA, USA) primary antibodies. Images were obtained by Zeiss microscopy (Carl Zeiss, Thornwood, NY, USA) and quantified using Image-Pro 4.5 software (Cybernetics, Silver Spring, MD, USA).

### 4.6. Terminal Transferase-dUTP-Nick-End Labeling (TUNEL) Assay

Apoptosis was measured using the TUNEL assay according to the manufacturer’s protocol (Roche Diagnostics, Mannheim, Germany). Briefly, after deparaffinization and rehydration, kidney sections were incubated with TUNEL reaction mixture for 60 min at 37 °C in a humidified dark chamber. Images were taken using a Zeiss ApoTome Axiovert 200M microscope (Carl Zeiss Microscopy).

### 4.7. Western Blot Analysis

Whole kidney protein was extracted with lysis buffer. After centrifugation (13,000 rpm, 4 °C, 15 min), the lysate was mixed with 5x sample buffer and heated at 95 °C for 6 min. Total protein concentrations were measured using Bradford methods (BioRad Laboratories, Hercules, CA, USA). Whole lysates were subjected to SDS-PAGE gel electrophoresis and transferred onto a polyvinylidene difluoride membrane (PVDF, GE Healthcare BioSciences Co., Piscataway, NJ, USA). PVDF membranes were blocked using 5% skim milk for 1 h at room temperature, and subsequently were incubated overnight at 4 °C with primary antibodies, such as anti-NGAL (1:1000; Abcam), anti-intercellular adhesion molecule-1 (ICAM-1, 1:1000; Santa Cruz Biotechnology), anti-inducible nitric oxide synthase (iNOS, 1:1000; Santa Cruz Biotechnology), anti-collagen I (1:1000; Southern Biotech), anti-collagen IV (1:1000; Southern Biotech), anti-fibronectin (1:1000; Santa Cruz Biotechnology), anti-alpha smooth muscle actin (α-SMA, 1:1000; Abcam), anti-p-nuclear factor kappa B (p-NF-κB, 1:1000, Cell Signaling Technology, Denver, MA, USA), anti-t-nuclear factor kappa B (t-NF-κB, 1:1000, Cell Signaling Technology), anti-p-ERK (1:1000, Cell Signaling Technology), anti-t-ERK (1:1000, Cell Signaling Technology), anti-glyceraldehyde 3-phosphate dehydrogenase (GAPDH, 1:1000; Sigma-Aldrich), and anti-heat shock 70 kDa protein 8 (HSC70, 1:1000; Santa Cruz Biotechnology). The blots were reacted with peroxidase-conjugated secondary antibodies (Vector Laboratories, Inc., Burlingame, CA, USA) and detected by enhanced chemiluminescent sensitive plus reaction (BioFX Laboratories, Inc., Owings Mills, MD, USA). The positive immunoreactive protein bands were detected by LAS-3000 film (FUJIFILM Corporation, Tokyo, Japan). Each blot density was normalized to GAPDH or HSC70 and compared with that of each control.

### 4.8. Quantitative Real Time Reverse Transcriptase Polymerase Chain Reaction 

Total cellular RNA was extracted with TRIzol reagent (Invitrogen, Carlsbad, CA, USA). Expression of mRNAs were measured by real-time PCR using a 20 μL reaction volume consisting of cDNA transcripts, primer pairs, and SYBR Green PCR Master Mix (Applied Biosystems, Carlsbad, CA, USA) with the StepOne^TM^ (Applied Biosystems). 18S was used as an internal control to normalize the genes. The primer sequences are shown in Table 1. 

### 4.9. Statistical Analyses 

All results are expressed as the mean ± standard error (SE). Analysis of variance (ANOVA) was used to assess the differences between multiple groups, followed by Fisher’s least significant difference (LSD) test. The level of statistical significance was set at *p*-values less than 0.05.

## Figures and Tables

**Table 1:** Primer sequences used for real time RT-PCR analysis.

Gene	Forward (5′ → 3′)	Reverse (5′ → 3′)
18S	CGAAAGCATTTGCCAAGAAT	AGTCGGCATCGTTTATGGTC
NGAL	GGCCAGTTCACTCTGGGAAA	TGGCGAACTGGTTGTAGTCC3
ICAM-1	CTTCCAGCTACCATGCCAAA	CTTCAGAGGCAGGAAACAGG
iNOS	GGCAGCCTGTGAGACCTTTG	CATTGGAAGTGAAGCGTTTCG
IL-6	AGTTGCCTTCTTGGGACTGA	TCCACGATTTCCCAGAGAAC
NOX1	AGCCATTGGATCACAACCTC	AGAAGCGAGAGATCCATCCA
NOX2	TGCACCATGATGAGGAGAAA	CCACACAGGAAAACGCCTAT
NOX3	ATTTCACTACCCCGTGAGCG	TCAGGCAGGCTCTGTGATTC

## Data Availability

Not applicable.

## Supplementary Materials

The following are available online at https://www.mdpi.com/article/10.3390/ijms22115667/s1, Figure S1: Effect of LJ-4459 on urinary excretion of adenosine. Figure S2: Effect of LJ-4459 on cell viability of mProx cells.
