# Supplementary material for: Dual Actions of A2A and A3 Adenosine Receptor Ligand Prevents Obstruction-Induced Kidney Fibrosis in Mice
Source: Int J Mol Sci. 2021 May 26;22(11):5667. doi: 10.3390/ijms22115667 (PMC8198234; doi:10.3390/ijms22115667)
Supplement: Supplementary file 1 [file ijms-22-05667-s001.zip › ijms-1205247-supplementary.pdf]

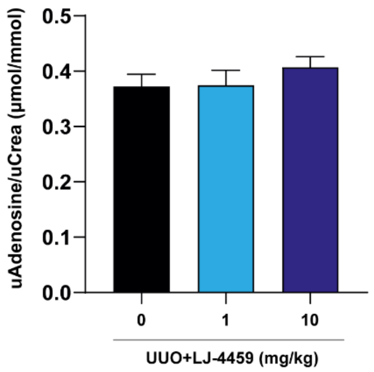

Supplementary Figure S1. Effect of LJ-4459 on urinary excretion of adenosine. Data are presented as mean ± SE of 7-8 mice.

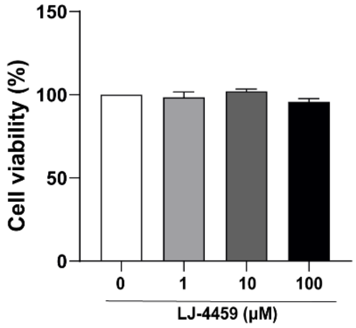

Supplementary Figure S2. Effect of LJ-4459 on cell viability of mProx cells. mProx cells were treated with different concentrations of LJ-4459, and cell viability was assessed by MTT assay. Data are presented as mean ± SE of 3 independent experiments.

Supplementary Methods

Measurement of adenosine

Urine was collected for 24 h and centrifuged at 3,000 rpm for 15 min at 4 °C. Adenosine was measured by using a fluorometric kit according to the manufacturer’s protocol (Abcam, Cambridge, MA, USA).

mProx Cell culture

The mProx cells were maintained in Dulbecco’s modified Eagle’s medium (DMEM; Invitrogen, Carlsbad, CA, USA) containing 10% fetal bovine serum (FBS; GIBCO by Life Technologies, Carlsbad, CA, USA), 100 U/mL penicillin, 100 μg/mL streptomycin, at 37°C in a humidified incubator with 5% CO_2.

MTT assay

Cell viability and toxicity were measured using MTT (3-[4, 5-dimethylthiazol-2-yl]-2, 5-diphenyl tetrazolium bromide) assay. The mProx cells were seeded at 4×10^4 cells/well in a 96-well plate and treated with LJ-4459 at the concentrations of 1, 10, and 100 μM for 24 h. At the end of treatment, the MTT solution was added to make a final concentration of 1 mg/ml, and the assay was performed according to the manufacturer's instructions (Sigma). The absorbance was measured at 570 nm by ELISA reader (ThermoFisher Scientific, Waltham, MA, USA).
